# Brain Dopamine–Clock Interactions Regulate Cardiometabolic Physiology: Mechanisms of the Observed Cardioprotective Effects of Circadian-Timed Bromocriptine-QR Therapy in Type 2 Diabetes Subjects

**DOI:** 10.3390/ijms241713255

**Published:** 2023-08-26

**Authors:** Anthony H. Cincotta

**Affiliations:** VeroScience LLC, Tiverton, RI 02878, USA; anthony_cincotta@veroscience.com; Tel.: +1-401-816-0525

**Keywords:** bromocriptine, dopamine, diabetes, circadian, Cycloset, neuroendocrine, suprachiasmatic nucleus, cardiovascular, metabolic syndrome

## Abstract

Despite enormous global efforts within clinical research and medical practice to reduce cardiovascular disease(s) (CVD), it still remains the leading cause of death worldwide. While genetic factors clearly contribute to CVD etiology, the preponderance of epidemiological data indicate that a major common denominator among diverse ethnic populations from around the world contributing to CVD is the composite of Western lifestyle cofactors, particularly Western diets (high saturated fat/simple sugar [particularly high fructose and sucrose and to a lesser extent glucose] diets), psychosocial stress, depression, and altered sleep/wake architecture. Such Western lifestyle cofactors are potent drivers for the increased risk of metabolic syndrome and its attendant downstream CVD. The central nervous system (CNS) evolved to respond to and anticipate changes in the external (and internal) environment to adapt survival mechanisms to perceived stresses (challenges to normal biological function), including the aforementioned Western lifestyle cofactors. Within the CNS of vertebrates in the wild, the biological clock circuitry surveils the environment and has evolved mechanisms for the induction of the obese, insulin-resistant state as a survival mechanism against an anticipated ensuing season of low/no food availability. The peripheral tissues utilize fat as an energy source under muscle insulin resistance, while increased hepatic insulin resistance more readily supplies glucose to the brain. This neural clock function also orchestrates the reversal of the obese, insulin-resistant condition when the low food availability season ends. The circadian neural network that produces these seasonal shifts in metabolism is also responsive to Western lifestyle stressors that drive the CNS clock into survival mode. A major component of this natural or Western lifestyle stressor-induced CNS clock neurophysiological shift potentiating the obese, insulin-resistant state is a diminution of the circadian peak of dopaminergic input activity to the pacemaker clock center, suprachiasmatic nucleus. Pharmacologically preventing this loss of circadian peak dopaminergic activity both prevents and reverses existing metabolic syndrome in a wide variety of animal models of the disorder, including high fat-fed animals. Clinically, across a variety of different study designs, circadian-timed bromocriptine-QR (quick release) (a unique formulation of micronized bromocriptine—a dopamine D2 receptor agonist) therapy of type 2 diabetes subjects improved hyperglycemia, hyperlipidemia, hypertension, immune sterile inflammation, and/or adverse cardiovascular event rate. The present review details the seminal circadian science investigations delineating important roles for CNS circadian peak dopaminergic activity in the regulation of peripheral fuel metabolism and cardiovascular biology and also summarizes the clinical study findings of bromocriptine-QR therapy on cardiometabolic outcomes in type 2 diabetes subjects.

## 1. Introduction

During the last century, including within the last three decades, the prevalence of cardiovascular disease (CVD) has been on the rise across the globe, currently afflicting an estimated 550 million people [1]. While genetic aberrations can account for various cardiovascular disorders, the breadth of this “recent” rise in global CVD burden cannot be attributed to any genetic shift within the worldwide population within this 100-year span. Rather, the rise in CVD prevalence across distinct populations of the world coincides most closely with the onset within these populations of the “westernized lifestyle” characterized predominantly by a **chronic shift** to a combination of high simple sugar [particularly including high fructose, sucrose]/high saturated fat [absence of mono- and polyunsaturated fats] diets, an increase in disruption to normal nocturnal daily sleep/wake architecture, artificial photoperiod exposure (including light at night), depression, and/or an increase in psychosocial stress [2,3]. More importantly, each of these environmental alterations has been well documented in preclinical and clinical studies to induce biochemical pathophysiological changes, including obesity, hypertension, insulin resistance, dyslipidemia, and/or systemic low-grade inflammation (collectively defined as/termed metabolic syndrome or cardiometabolic syndrome) that precipitate CVD [4,5,6,7,8,9,10,11,12,13,14,15,16,17,18,19,20,21,22,23,24,25]. Each of these environmental perturbations is processed, interpreted, and acted upon largely by the central nervous system (CNS) to orchestrate whole-body physiological responses. An ancient endogenous environmental response system in vertebrates is the CNS biochemical machinery that evolved to record and anticipate natural recurring seasonal changes in the environment (such as daily and seasonal changes in photoperiod, temperature, rainfall, food availability and quality) to maximize survival potential against ensuing environmental challenges [26,27,28,29,30,31,32,33]. The components of this CNS anticipatory response system define the framework of the CNS biological clock circuitry. Vertebrate species, from teleosts to mammals in the wild from around the globe, exhibit distinct seasonal changes in metabolic, reproductive, immunological, and behavioral physiology [26,27,28,29,30,31,32,33]. Respecting metabolism, animals in the wild become obese and insulin resistant in preparation for an ensuing season of low/no food/glucose availability and at the end of this low food availability season, they spontaneously become lean and insulin sensitive once again; seasonal shifts that transpire under laboratory conditions of unchanging food supply and photoperiod (i.e., it is an endogenous mechanism) (reviewed in [26,27]). The seasonal insulin-resistant state facilitates increased hepatic glucose output that under peripheral insulin-resistant conditions is more readily shunted to the brain, which has a near-absolute requirement for glucose as an energy source while the peripheral tissues (e.g., muscle) utilize lipid stores for energy supply further driving/maintaining muscle insulin resistance. An appreciation of the nature of this evolutionarily well-preserved endogenous biological clock system and its response to aforementioned environmental stresses of altered diet, sleep/wake architecture, and psychosocial stress are critical in understanding (a) how such stresses alter cardiometabolic function (induce pathology) and (b) the physiological mechanisms contributing to/driving the effectiveness of circadian-timed bromocriptine-QR therapy for cardiovascular disease. Therefore, this review must begin with an examination of the critical CNS circadian clock elements and functions observed to regulate daily and seasonal metabolism in vertebrates, including man.

## 2. Temporal Synergisms of CNS Circadian Neural Oscillations Dictate Physiological Status

Human observations and documentation of annual cycles of physiological activities (e.g., metabolic, behavioral [migratory], reproductive status) among vertebrate species in the wild appear to date back far beyond antiquity [34], yet we are only recently beginning to understand the mechanisms operative in the genesis of these precisely timed, marked changes in vertebrate physiology. A multitude of seminal studies by the laboratory of Albert H. Meier, initiated over 50+ years ago, were the first to identify both a vertebrate circadian physiological response to any molecule (in this case, the hormone prolactin) administration [35] and specific roles for temporal interactions of CNS neuronal circadian activities in the regulation of seasonal vertebrate physiology (reviewed in great detail in [26,27,36,37,38]). These initial studies found that the phase (time of day) relationship between the circadian peak (acrophase) in plasma prolactin and corticosteroid hormone differed in seasonal obese and lean animals and that merely mimicking the circadian phase relationship of these hormones of a particular season (by their exogenous injection at the appropriate times of day *under constant light conditions* for approximately 7–14 days) was capable of inducing that seasonal metabolic condition irrespective of the actual time of year [39,40,41]. For example, it was demonstrated to be possible to induce the seasonal lean, insulin-sensitive condition in obese, insulin-resistant animals during the seasonal obese, insulin-resistant time of year by mimicking the plasma circadian acrophase relationship of these hormones (via hormonal injection at the appropriate times of day) of the lean, insulin-sensitive season [and also vice versa] regarding shifting from lean to obese seasonality [42]. This temporal synergism of circadian neuroendocrine rhythms responsible for the manifestation of seasonal metabolic status was demonstrable across representative species of all the major vertebrate classes [41], highlighting the positive selection force for its preservation across more than 400 million years of evolution [26]. As an example of the power of this circadian system in regulating seasonal physiology, sterile southward-bound migratory birds of Fall could be physiologically “reset” to fertile, reproductively active, northward-bound migratory birds (of Spring) by a short 7-day duration of circadian-timed prolactin and corticosteroid hormone treatment at times of day that mimic the circadian peaks in the plasma levels of Spring birds [37]. In migratory fish, this same methodology is capable of resetting not only metabolism but also salinity preference, able to convert fish from seasonally saltwater living to seasonally freshwater living (and vice versa) within days [36] (try tossing a saltwater fish into fresh water and observe its health to appreciate this dramatic response to circadian resetting). Moreover, the effects of this short-term neuroendocrine “resetting” intervention (usually 7–14 days) *persisted for many months* following the termination of the treatment [37,42]. In other words, the animals were shifted from one particular seasonal physiology to another and then remained there out of sync with the real world for several months thereafter. Since animals will exhibit an annual cycle of physiology under constant light conditions and this temporal synergism “resetting” of the annual cycle method also is effective under constant light (and food supply) conditions, something *endogenous* must be “driving” this control of seasonally shifting metabolic state independent of photoperiod. For instance, note that the photoperiod of 12 h of light is the same in Fall and Spring times in temperate zones of the earth, yet studied vertebrates (e.g., mammals) at these two seasons are markedly different physiologically, exhibiting essentially opposite reproductive behavior, reproductive capacity, and migratory orientation. Furthermore, the annual cycle of whole-body physiology (including metabolism, reproduction and behavior) can manifest without any change in the photoperiod during certain times of the year (e.g., obese, insulin-resistant animals of winter will pass into the spring lean, insulin-sensitive condition even if maintained on the same short [10 h]) daily photoperiods of winter) [43]. However, at certain checkpoint times of the year, the change in photoperiod is required to advance/change the physiological status to that of the next season (see discussion below on circadian regulation of seasonality). A critical question is: Within this chronobiological framework, how and where are the circadian-timed injections of prolactin and corticosteroid hormone working to reset the annual cycle of (metabolic) physiology and how does disruption to this clock system precipitate CVD?

## 3. CNS Circadian Clock Mechanisms Generating Seasonal Physiology

The CNS of animals is equipped with the ability to determine and respond to photoperiod length (the onset of light sets a daily rhythm of photosensitivity and if light is present during the photosensitive phase of the day (e.g., at 14 h after light onset, [long daily photoperiod]) it produces a different physiological response than if light is not present during this photosensitive period (e.g., as would occur on 10 h [short] daily photoperiods), a physiological phenomenon termed *photoperiodism* [26,27]. Additionally, the CNS is equipped with the ability to change its interpretation of the same photoperiod at different times of the year, based largely upon the number of days the CNS is exposed to that photoperiod—i.e., after a certain number of days has passed on a given photoperiod, the CNS response to that photoperiod changes (a delayed response to photoperiod)—a physiological phenomenon termed *seasonality* [26,27]. The interaction of photoperiodism and seasonality functions of the CNS produce the annual cycle of physiology in vertebrates. This is an exceedingly important point to understand to appreciate CNS circadian clock control of physiology, so we will provide an example of its operation in the seasonal Syrian hamster. Female Syrian hamsters are naturally born in spring on long (~12.5–14 h) daily photoperiods and mature to adulthood during the spring and summer. At least ten weeks of exposure to such long daily photoperiods is required for the animals to become sensitive to the effects of subsequent short daily photoperiods of fall and winter (<12 h/day of light) to induce reproductive regression (they become essentially sterile during this time of year when held on short daily photoperiods) and severe insulin resistance and obesity. If maintained on long daily photoperiods through Fall and Winter, this annual physiological shift is significantly attenuated but not eliminated (photoperiodic effect). Following such exposure to short daily photoperiods for approximately 14 weeks, the animals become refractory to the short daily photoperiod effects on reproduction and metabolism (even while held on the same short daily photoperiod), and the animals spontaneously become reproductively fertile and active and the obese, insulin-resistant state is reversed (seasonality effect). Exposure to long daily photoperiods is then required for about 10 weeks to initiate another annual cycle [42,43,44,45].

These neural environmental processing events occur within the brain clock system of which the suprachiasmatic nucleus (SCN) is the primary pacemaker receiving both photic and non-photic information (from the retinohypothalamic tract and other CNS/peripheral inputs, respectively), but this clock system also includes several other brain areas including the supramammillary nucleus, the lateral habenula, the amygdala, and striatum with which the SCN communicates [46,47,48,49,50,51]. The actual biochemical machinery that runs the clock within the neuronal cells of this clock network (and all cells of the body) has been well delineated in the past several years and is the topic of well-researched reviews [52,53]. Importantly, this evolutionary preserved cellular clock machinery is operative in nearly every cell of the mammalian body. It is a primary role of the CNS clock network, particularly the SCN, to modulate the expression of clock and clock-controlled genes in the peripheral tissues for the temporal synchronization of cellular biochemical activities in turn for the production of tissue-level functions and temporal integration of organ systems biology to generate a viable biological organism (see discussion below). Provided here is a very brief and basic summation of the salient components of this cellular clock machinery.

Twenty-four-hour oscillations in cellular gene expressions are manifested by transcriptional-translational feedback loops within core clock genes wherein the clock gene products BMAL1 and CLOCK heterodimerize in the cytosol over time to a certain accumulated level (similar in concept to a filling hourglass mechanism) before translocating back to the nucleus to bind to DNA E-box elements of promoter regions for *PERIOD* (*PER1/2/3*) and *CRYPTOCHROME (CRY1/2)* gene transcription, heterodimer protein products of which upon appropriate accumulated cytosolic level over time also translocate back to the nucleus to bind to the BMAL1/CLOCK complex to block its transcriptional activity—a circular process that takes approximately 24 h to complete. In a second feedback loop, the nuclear BMAL1/CLOCK heterodimer also activates the transcription of REV-ERBα/β proteins, which also feedback from the cytosol upon appropriate level accumulation over time to repress nuclear activation of *BMAL1/CLOCK* gene expression in a 24-h oscillatory fashion by blocking circadian RORα/β/γ protein activation of *BMAL1* and *CLOCK* transcription. Nuclear PER, CRY, and REV-ERB protein levels are reduced by a variety of posttranslational modifications, including phosphorylation and enzymatic degradation (themselves circadian in nature, ultimately regulated by the BMAL1/CLOCK-PER/CRY -ROR/REV-ERB interaction complexes), thus allowing for the initiation of another 24-h *BMAL1/CLOCK* transcriptional cycle under circadian ROR activation. Circadian *ROR* expression is itself regulated by BMAL1/CLOCK via its circadian transcriptional control of *DBP*, an activation transcription factor for *ROR* (and several other genes), while REV-ERB provides circadian transcriptional control of *NFIL3*, another transcription factor that represses *DBP* activity. These interlocking feedback loops provide multiple layers of circadian core clock gene regulation. Moreover and most importantly, since the BMAL1/CLOCK, PER/CRY, ROR/REV-ERB, and DBP/NFIL3 monomer and heterodimers act as transcription factors for the transcription of many thousands of genes, the circadian expression (daily acrophase [time of daily peak function] and amplitude) of *BMAL1* and *CLOCK* and these other core clock genes regulates the daily temporal organization of nearly all cellular biochemical reactions essential for life [52,53]. The core clock genes are responsive to changes in the cellular environment, such as pH, metabolites, xenobiotics, hormones, growth factors, nutrients, microRNAs, and more [54,55]. An important question is: What can set the acrophase and amplitude of the circadian peak expression of *BMAL1/CLOCK-PER/CRY-ROR/REV-ERB- DBP/NFIL3* gene transcription activities in particular cells, especially within the SCN neuronal pacemaker system, to modify their regulation of particular circadian biological functions, such as neuronal output activity? In other words, what is regulating the circadian expression of the core clock genes in the CNS clock pacemaker system of the body to regulate its circadian output control of whole-body (circadian) physiology?

**Figure 1:**
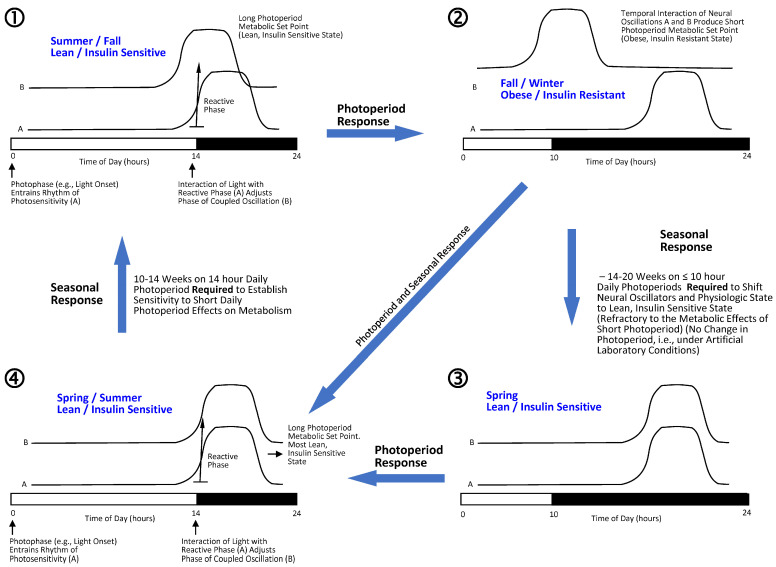
The blueprint for interactions of photoperiodic and seasonal clocks generates the annual cycle of physiology (e.g., metabolism) in vertebrates under natural conditions. Photoperiodic effects on physiology are manifested by the changing phase relationships of at least two CNS circadian neuroendocrine oscillations (termed internal coincidence). One oscillation (A) is directly entrained by the daily photoperiod (e.g., light onset). The second oscillation (B) is loosely coupled with oscillation A in different phase relationships as a function of daily photoperiod length (i.e., whether or not light coincides with a reactive [i.e., sensitive] phase of oscillation A) (transition from Summer/Fall lean, insulin-sensitive metabolic condition 1 to Winter obese, insulin-resistant condition 2 in the figure). The differing phase relations of these interacting circadian neural oscillations produce differing neuroendocrine output effects downstream on the body as a function of their phase relationships. Temporal interactions of CNS circadian neural oscillations also manifest a seasonal clock that regulates physiology. After a certain number of days on a particular seasonal photoperiod (e.g., short [≤ 10 h] photoperiods of Winter), an internal day-counting mechanism involving melatonin interactions with the CNS clock circuitry elicits a temporal shift in CNS circadian oscillations *while animals are held on the existing photoperiod* to manifest a resultant new seasonal condition on the same photoperiod (transition from Winter metabolic condition 2 to Spring metabolic condition 3 in the figure). Additionally, at certain seasonal checkpoints (e.g., after >/= 14 weeks on Winter short [</= 10 h] daily photoperiods), a change from short daily photoperiod to long daily photoperiod (>/= 14 h) of Spring/Summer (transition from Winter 2 to Spring/Summer 4 condition on the figure) is required for an approximate 10–14 week period to re-establish sensitivity (physiological responsiveness) to the next year’s Winter daily photoperiod (seasonal effect) (transition from Spring/Summer condition 4 to Summer/Fall condition 1 on the figure). ***Importantly, these circadian neural oscillations that govern whole body physiology (e.g., metabolism) may be phase and/or amplitude shifted without any change to the photoperiod by environmental stressors or by pharmaceutical agents to thus impact physiology/pathology.*** Updated from [26]; Copyright 1996 American Diabetes Association. From Diabetes Reviews®, Vol. 4, 1996; 464–487 [26]., updated with permission.

The fact that the annual cycle of physiology is reprogrammable by circadian-timed prolactin and corticosteroid hormonal administrations, as described above, suggests that both photoperiodism and seasonality are each the result of changing acrophase (time of daily peak function) relations of *at least* two CNS circadian neural oscillations, a postulate termed *internal coincidence model of clock function or temporal synergism* of CNS circadian oscillators. Regarding photoperiodism, the onset of light sets a rhythm of photosensitivity to the light, and if light is present later during a photosensitive time of day, it acts on the CNS to adjust a second circadian rhythm of neural activity into a particular acrophase relationship with itself, while if light is not present during this photosensitive time of day, it acts on the CNS to adjust the second circadian rhythm of neural activity into a different acrophase relationship with itself (i.e., an adjustable rhythm dependent upon photoperiod). Regarding seasonality, after a certain duration of days on a particular photoperiod has passed, *then without any change of photoperiod*, the second adjustable circadian neural oscillation changes its acrophase relationship with the neural oscillation set by the photoperiod (e.g., a shift from short photoperiod sensitivity that produces a particular physiology to short photoperiod refractoriness that no longer produces that physiology) (see refs. [26,27] for fuller description including regulation of photoperiodism and seasonality). This automatic shift requires a mechanism for *counting the days on a particular photoperiod*, and available evidence indicates that the duration and acrophase of the daily CNS melatonin rhythm are involved in providing this function [56]. In this construct, at least two environment-responsive CNS circadian oscillators change their acrophase relationship during the year to drive seasonal physiology (see Figure 1 for schematics of circadian neural temporal synergism manifestation of photoperiodism and seasonality that generate the annual cycle of vertebrate physiology).

**Figure 2:**
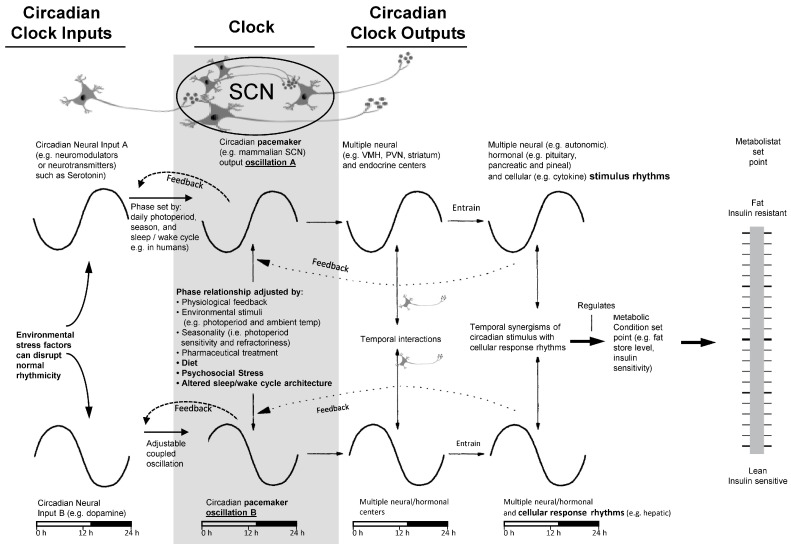
Circadian Metabolistat Framework for Regulation of Metabolism. The metabolistat is thought to have circadian components that include the primary circadian pacemaker (i.e., mammalian SCN) and a hierarchy of secondary (e.g., VMH) centers. Circadian temporal interactions of multiple neural oscillators within the SCN produce temporal interactions of circadian output signals to the autonomic and endocrine centers that, in turn, produce a myriad of circadian stimulus signals to the (metabolic) tissues of the body. These temporal interactions of circadian output signals to the autonomic and endocrine centers also entrain tissue response rhythms (circadian receptor and post-receptor expressions for hormones and neurotransmitters). The circadian interactions of multiple circadian stimuli and response rhythms at the metabolic tissues (cellular level) determine the tissue metabolic output, e.g., increased or decreased hepatic lipid synthesis. SCN = Suprachiasmatic Nucleus; VMH = Ventromedial Hypothalamus; PVN = Paraventricular Nucleus. Updated from [26]; Copyright 1996 American Diabetes Association. From Diabetes Reviews®, Vol. 4, 1996; 464–487 [26]., updated with permission.

The photoperiod is a dominant time setter (zeitgeber) input signal to the CNS clock system from the retina to the SCN (the retinohypothalamic tract), as mentioned above. However, this circadian circuitry also receives input from a multitude of internal non-photic cues (e.g., other CNS and peripheral nervous system (PNS) centers, paracrine factors, humoral factors, and nutrients) [46,47,48,49,50,51,57,58,59,60]. Each of the photic and non-photic neuronal input circuits to the clock can be markedly modified by several external environmental factors, provided these factors persist chronically, and they include alteration to diet (high saturated fat/simple sugar diet), psychosocial stress (including depression and anxiety), and altered sleep/wake architecture (e.g., interrupted sleep, too long or short sleep duration, shift work, light at night) [58,59,60,61,62,63,64,65,66,67,68,69,70,71,72,73,74,75]. It now appears that it is the temporal interaction of these non-photic cues with the photoperiod-sensitive cues that ultimately determine the neuronal nature of the clock output oscillations (i.e., clock “interpretation” of and response to the photoperiod) that govern clock organization/regulation of whole-body physiology. Each CNS clock oscillator output entrains separate multiple neural and hormonal downstream circadian expressions (e.g., CNS, hypothalamic, autonomic activities and endocrine functions in various tissues, such as the pituitary, pancreas, and gut). The temporal circadian interactions of such CNS clock oscillator systems at the cellular level (e.g., stimulus rhythms [neurotransmitters, hormones, and/or humoral factors] interacting with response rhythms [cellular receptor/signal transduction activity rhythms]) govern cellular gene expression and biochemistry to generate a particular circadian cellular physiology (see below for exemplary description). This circadian cellular entrainment then generates synchronized tissue-level physiology and manifests a whole-body physiology that is temporally organized within organ systems internally and with the organism’s cyclic environment (Figure 2).

Available evidence suggests that environmental stress factors (e.g., Western diet, psychosocial stress, altered sleep/wake architecture) can shift clock control of the circadian metabolistat to induce insulin resistance syndrome while adjustment of the altered circadian metabolistat, as with bromocriptine and other neurotransmitter-affecting drugs, can be an effective treatment aimed at the cause rather than the symptoms of many physiological disorders. The SCN is the primary mammalian circadian pacemaker for the body, but the CNS clock includes its direct and polysynaptic connections with other clock centers (e.g., lateral habenula and others).

By way of example of how this system is posited/understood to operate (derived from available experimental evidence) respecting circadian regulation of peripheral metabolism, one clock oscillation drives a circadian rhythm of liver lipogenic responsiveness to insulin [76,77,78] while another oscillation drives a rhythm of plasma insulin level [79] and the acrophase relationship between the two determines the magnitude of lipogenesis. The maximum effect occurs when the stimulus and response rhythms are in phase, while a gradation of lesser effects manifests as the acrophases of the two rhythms move apart [76,77]. Expanding this singular example of hepatic lipogenesis to multitudes of cellular stimuli and response rhythms generates the living cell biology. For example, regarding hepatic lipogenesis, it was found in the Syrian hamster that the amplitude in the acrophase of the circadian rhythm of hepatic lipogenic responsiveness to insulin is set by the amplitude of the circadian peak in plasma prolactin during the daily peak in liver lipogenic responsiveness to prolactin [77,80]. Thus, temporal synergisms among multiple circadian biological activities generate the level of daily lipid production in liver tissue. Indeed, the first demonstration of cellular clock mechanisms operating autonomously outside the CNS but responsive to circadian input signals was of hepatocyte circadian lipogenic activity in the cell culture [81]. These same studies highlighted the important role of the CNS clock system in organizing cellular circadian rhythms of biochemistry within the tissue for the generation of tissue-level circadian biological activities. At the whole animal level, this complex circadian system organizes multiple CNS circadian neuronal activity interactions to manifest increased hepatic lipogenic responsiveness to insulin coincident with the feeding time of day and increased adipose lipolytic activity coincident with the fasting (sleeping) period of the day [43,76,78]. The interaction of these CNS oscillators can, thus, regulate the amplitude of daily lipogenic and lipolytic activities along with a host of other metabolic (glucoregulatory) and behavioral (feeding) events in multiple tissues to establish body composition and glucose metabolism homeostasis set points so the animal (human) physiology is synchronized temporally within its tissues and with its cyclic environment [26,27]. Animals defend very specific body composition set points during different times of the year irrespective of excess food availability during the lean season or limited food availability (or even surgical adipose removal) during the obese season, and available evidence suggests that this is a result of such CNS temporal circadian neuronal interactions (reviewed in [26,27]). This circadian regulatory framework for control of body fat store level is operative across multiple biochemical physiology systems, including control of fuel metabolism, reproduction, and behavior that have been investigated in a variety of vertebrate species [26,36,37,38,39,40,41,42].

## 4. CNS Circadian Dopaminergic Neuronal Input Modulation of CNS Clock Output Control of Cardiometabolic Status

Based upon such observations of the circadian organization of cellular and organismal-level biology, it was therefore surmised that the timed daily injections of prolactin and corticosteroid hormone that were capable of resetting the annual cycle of metabolism in vertebrates as described above were acting on clock neurons within the CNS to “reset” the acrophases of at least two pacemaker oscillators of an endogenous clock mechanism constructed of multiple circadian neural oscillators governing whole-body physiological status as a function of the temporal (acrophase) relationship of their circadian interactions. Each neural oscillation responsive to prolactin or corticosteroid drives several downstream parallel target oscillation(s) (e.g., within other CNS nuclei [e.g., hypothalamic, limbic] or endocrine organs) that, in turn, send circadian neuroendocrine information to the periphery (e.g., liver) that interact temporally with circadian rhythms of responsiveness to these circadian neuroendocrine oscillations to manifest cellular biochemistry as exampled above for hepatic lipid metabolism. Although plasma prolactin and corticosteroid act directly on multiple peripheral target tissues, it was proffered that it is their effect on the CNS that sets these circadian events in motion, including responsiveness to themselves in the periphery [26,27,41].

Prolactin is a strong stimulus for dopamine synthesis via stimulation of tyrosine hydroxylase (TH) synthesis and activity [82], and corticosteroid hormone is a strong stimulus for serotonin synthesis via stimulation of tryptophan hydroxylase activity [83], so subsequent seasonal resetting studies were conducted simply replacing the circadian-timed injection of prolactin with L-DOPA (the substrate for TH and dopamine synthesis) and circadian-timed injection of corticosteroid hormone with 5-hydroxy-tryptophan (5-HTP, the precursor for serotonin) to test for a neuronal basis of these hormonal effects. In fact, across representative species of major vertebrate classes from fish to mammals, such circadian timed L-DOPA and 5-HTP injections did reset the annual cycle of physiology, analogous to the response to circadian timed injections of prolactin and corticosteroid hormone, respectively, as expected [84,85,86,87]. Subsequent neurophysiological studies identified the hypothalamic SCN as a primary center within the CNS as a site where the endogenous dopamine and serotonin activity rhythms operate to control SCN circadian output signals to the tissues and organs of the body to establish physiological status, such as metabolic condition [88,89,90,91,92,93,94,95] (Figure 2).

**Figure 3:**
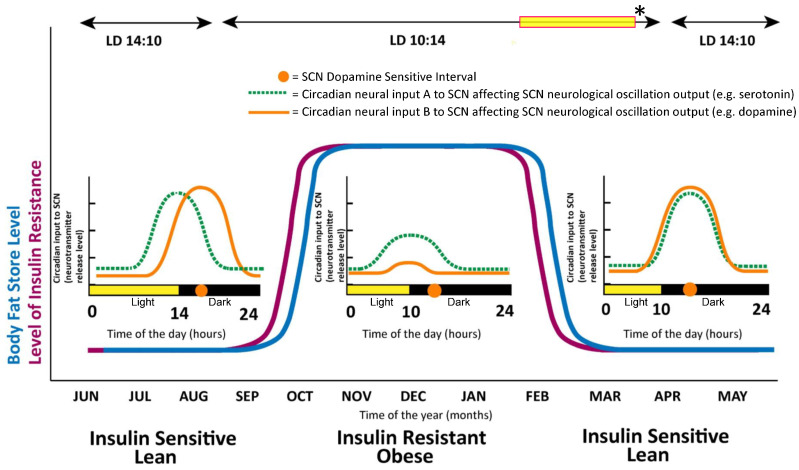
Temporal Interactions of Afferent Circadian Neural Inputs to the Biological Clock Circuit Pacemaker (SCN) Manifest the Annual Cycle of Metabolism. Circadian Organization of the Annual Cycle of Metabolism of a Representative Mammalian Species. Afferent input signals to the SCN (e.g., dopaminergic and serotonergic) shift their circadian temporal organization seasonally as part of the annual clock system in vertebrates to program changing seasonal output signals from the SCN to the neuroendocrine axis that, in turn, manifest different seasonal metabolic conditions (e.g., obese, insulin resistant or lean, insulin-sensitive states). * The yellow bar signifies the occurring seasonal shift in metabolism even without a seasonal change in photoperiod (i.e., under laboratory conditions). Updated from [26]; Copyright 1996 American Diabetes Association. From Diabetes Reviews®, Vol. 4, 1996; 464–487 [26], updated with permission.

The circadian rhythms (phase and amplitude) of dopamine and serotonin release at the SCN area differ markedly in seasonally lean, insulin sensitive and obese, insulin-resistant animals held on the same daily photoperiod at the same time of year (i.e., animals refractory to or sensitive to the effects of short daily photoperiods on induction of the obese, insulin-resistant condition) [93] (Figure 3).

Respecting the clock regulation of metabolism, the circadian peak of dopaminergic input activity to the SCN in seasonally lean, insulin-sensitive animals is severely attenuated in obese, insulin-resistant animals, and the serotonin rhythm is phase advanced and diminished [93]. Moreover, it appears that the dopaminergic input to the SCN can influence the serotonin rhythm therein. These observations led us to studies investigating a possible cause-effect relationship between attenuation of the circadian peak in SCN dopaminergic input activity and induction of the insulin-resistant, glucose-intolerant state. Destroying the dopaminergic neurons that input to the SCN area in lean animals by site-targeted, selective neurotoxin administration is sufficient to manifest the obese, insulin-resistant/glucose intolerant condition within weeks without alteration of food consumption in seasonal hamsters, demonstrating the high placement of the CNS clock circuit on the hierarchy of peripheral metabolism control [92]. Importantly, in laboratory rats that were sensitive to the obesogenic effects of a high-fat diet (60% of calories from fat), a similar attenuation of the circadian peak of dopaminergic input activity to the SCN was observed upon such high-fat feeding [94], and selective neurotoxin destruction of these dopaminergic neuronal fibers in rats resistant to the obesogenic effects of high fat diet-induced the obese, insulin resistant, glucose intolerant condition [95]. Moreover, such SCN dopaminergic neurotoxin intervention also induced a chronic activation of the sympathetic nervous system (SNS), hypertension, and elevated resting heart rate—i.e., in total—cardiometabolic syndrome [95]. A major site of circadian dopaminergic neuronal input to the SCN is the hypothalamic supramammillary nucleus (SuMN), a center with modulatory roles in reward perception, feeding, and peripheral fuel metabolism, and high-fat diet feeding markedly attenuates the circadian peak of dopamine synthesis at this center [95]. However, circadian-timed pharmacological stimulation of the SuMN at the daily peak of its dopaminergic activity reverses high-fat diet-induced insulin resistance and obesity. Furthermore, pharmacological attenuation of the SuMN output activity (particularly to the SCN) induces marked insulin resistance and obesity among animals held on a regular chow diet [96]. Additionally, administration of dopamine directly to the SCN of high fat fed—obese, insulin-resistant animals for just a few days at the time of its circadian peak in lean, insulin-sensitive animals was capable of reversing the effect of the high-fat diet on cardiometabolic syndrome [94]. Importantly, such dopamine administration to the SCN at times outside the circadian peak window of dopamine input in lean insulin-sensitive animals was without effect on cardiometabolic disease. These studies, in total, define a prodigious role for the circadian dopaminergic input modulation of CNS clock circuitry in the regulation of cardiometabolic health (and disease). Since our early studies of SCN control of peripheral fuel metabolism, a multitude of studies has demonstrated its critical role in this regard [97,98,99,100,101,102]. As mentioned above, SCN neuronal activities, directly and indirectly, modulate several metabolic control centers in the CNS, particularly the hypothalamus, mesolimbic system, and brain stem, to regulate autonomic and endocrine functions that control peripheral fuel metabolism. The SCN neuronal output modulates autonomic balance and endocrine input—to the liver to regulate fasting and postprandial glucose levels [103,104,105,106,107] and lipid balance [108]—to the adipose to regulate lipid balance [109]—to the pancreas to regulate insulin secretion [110]—to peripheral tissues to regulate insulin-stimulated glucose disposal [106,111]—to the vasculature and heart to regulate cardiovascular biology [112,113,114,115,116,117], and—to the immune system to regulate immune sterile inflammatory status that in turn modulates metabolism and cardiovascular biology [118,119,120,121,122].

To review at this point, the composite of the above-described findings then led to the following postulate regarding the circadian organization of whole-body physiology: At least two CNS endogenous pacemaker circadian oscillations responsive to environmental and internal feedback cues from the body, one being dopaminergic and one being serotonergic, interact temporally to modulate the circadian phase and amplitude of clock gene expressions within the CNS clock circuit that regulates circadian neuronal output control of the neuroendocrine axis to organize circadian activities within the peripheral tissues to generate specific physiology. This postulate requires (a) the existence of multiple independent neuronal circadian oscillations within the SCN (and also including within non-SCN circadian centers) [123,124,125,126,127,128,129] and (b) clock modulation of peripheral circadian stimulus and response rhythms within tissues of the body; both biological functions which have been confirmed [48,55,104,130,131].

## 5. SCN Output Circuits Controlling Metabolism and Cardiovascular Health

Inasmuch as the SCN communicates with the ventromedial hypothalamus (VMH) and hypothalamic paraventricular nuclei (PVN), which are critical hypothalamic centers for regulation of glucose homeostasis, body composition, feeding control, and autonomic regulation [27,131,132,133,134,135,136,137], we conducted a series of studies to delineate the neurophysiologic profile of these sites in seasonal SCN circadian peak eu-dopaminergic—lean, insulin-sensitive versus seasonal SCN circadian peak—hypodopaminergic obese, insulin-resistant animals. It was found that norepinephrine (NE) and serotonin (S) input to the VMH of seasonally obese insulin-resistant animals was markedly increased relative to seasonally lean, insulin-sensitive animals [138]. Moreover, studies from several laboratories demonstrated that the increased noradrenergic activity at the VMH was demonstrable across a wide variety of animal model systems of the obese, insulin-resistant state, including seasonal insulin resistance, high-fat diet feeding, leptin deficiency, leptin receptor attenuation, lethal yellow (A^y^/a) mice with inhibited hypothalamic αMSH, genetic-based insulin resistant, hypertension (SHR rat) and adult offspring of both pregnancy malnutrition and hyperinsulinemia mothers (reviewed in [27]). Further studies indicated that the responsiveness to norepinephrine within VMH neurons was also markedly increased in obese, insulin-resistant animals [139,140], providing a potential framework for sustained simultaneous increased noradrenergic stimulus and response systems in the VMH of obese, insulin-resistant animals, suggesting neuronal plasticity designed/evolved to potentiate the obese, insulin-resistant state. Importantly, administration of norepinephrine to the VMH of lean, insulin-sensitive animals to raise its VMH area level to that observed in obese, insulin-resistant animals induced the full cardiometabolic syndrome (including leptin resistance) within days without alteration in feeding, demonstrating the cause–effect relationship between increased VMH noradrenergic activity and cardiometabolic syndrome [141,142]. Supplementation of serotonin to the VMH norepinephrine administration exacerbated this norepinephrine response [141]. Moreover, the hyperinsulinemia induced by such VMH norepinephrine activity was found to feedback centrally to maintain such stimulated norepinephrine release at the VMH [143], closing/perpetuating a cardiometabolic syndrome precipitating loop, independent of feeding. Of additional note, the VMH is a prominent CNS fuel sensing center, wherein increases in local VMH levels of FFA or glucose (similar to post-meal levels) induce a VMH response that facilitates peripheral glucose uptake [144,145,146,147]. Norepinephrine administration to the VMH was found to block this VMH response to FFA and glucose, thereby facilitating glucose intolerance and, importantly, such administration also simultaneously elevated blood pressure [148,149,150,151]. It is critical to note that high-fat diet feeding both reduces the circadian peak in dopaminergic input activity to the SCN and resultantly elevates VMH noradrenergic activity [94], and this effect in and of itself is capable of attenuation of VMH fuel responsiveness, leading to decreased peripheral glucose disposal [94,148]. This body of work may be summarized as follows: An attenuation of the circadian peak dopaminergic activity at the CNS clock circuitry programs an activation of noradrenergic and serotonergic input activities at the VMH, which initiates multiple downstream neuroendocrine events leading to cardiometabolic syndrome (hypertension, obesity, insulin resistance, glucose intolerance, dyslipidemia), largely independent of alteration in feeding behavior. A similar circumstance regarding circadian dopaminergic SCN input regulation of SCN-directed metabolism was found to preside within the PVN as well, as described below.

**Figure 4:**
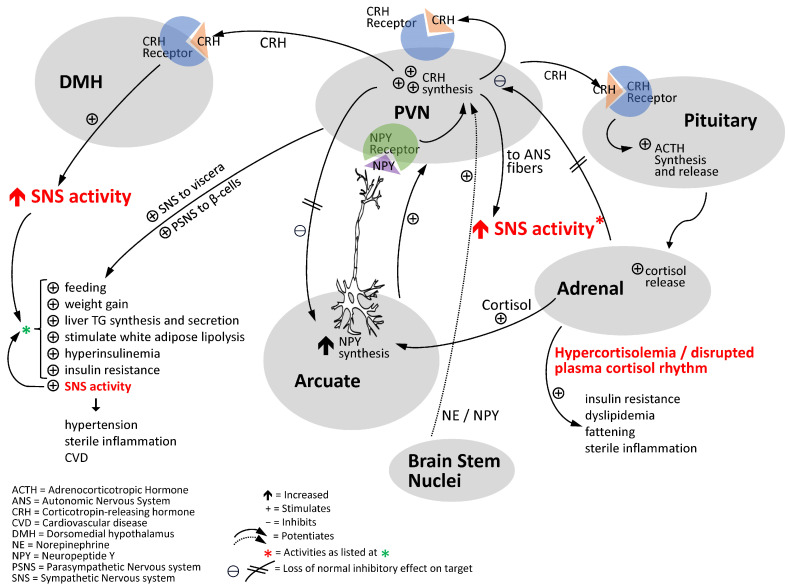
Interacting Positive Feedback Loops between Elevated PVN NPY and CRH Potentiating Insulin Resistance Syndrome. Diminished circadian peak dopaminergic input to the clock pacemaker (SCN) stimulates chronic NPY synthesis and release to the PVN and increases PVN CRH release. PVN NPY release stimulates feeding, hyperinsulinemia (via parasympathetic stimulation of the B cell), hepatic triglyceride synthesis, hypertension and SNS activation, which itself also potently potentiates insulin resistance syndrome. Importantly, NPY also stimulates PVN CRH synthesis and release, which potentiates insulin resistance syndrome via activation of SNS tone and downstream cortisol stimulation. Moreover, cortisol also feeds back to stimulate PVN NPY synthesis and release. The interlocking positive feedback loops between NPY and CRH keep their peripheral effects of dysmetabolism maintained long-term.

The hypothalamic PVN is another critical physiological control center in the CNS with major regulatory roles in feeding, glucose and lipid metabolism, autonomic balance, activation of the hypothalamic–pituitary–adrenal (HPA) corticosteroid hormone secretion axis, whole-body water/electrolyte balance, and reproduction [131,132,133,134,135,136,137]. Like the VMH, PVN activity is responsive to circadian dopaminergic modulation, and the PVN receives strong direct and indirect innervation from the SCN, which modulates both PVN autonomic and endocrine output signals to regulate hepatic glucose metabolism [131,152]. As it relates to the present discussion on cardiometabolic disease, neuropeptide Y (NPY) input to the PVN from the arcuate nucleus and brain stem and corticotropin-releasing hormone (CRH) from PVN neurons to other brain areas play pivotal roles in the regulation of metabolism. The neurophysiological interactions between hypothalamic NPY and CRH are complex and dependent upon the prevailing local and organismal level biology [153,154], however in the circumstance of the chronic stress-related obese, insulin-resistant state, a unique neuropathological interaction appears to preside. Accumulating evidence suggests that both PVN NPY and CRH levels/activity are often simultaneously elevated and participate in the development of the obese, insulin-resistant, hypertensive state as follows (reviewed in [27,155,156]). Elevated PVN NPY activity is a potent stimulus for hyperinsulinemia, hyperlipidemia, overfeeding, fattening, and chronically elevated SNS tone [27,132,155,157,158,159,160,161]. Chronic PVN CRH activity not only activates the HPA axis to drive hypercortisolemia, itself a potent stimulus for insulin resistance and fattening [155,162,163,164,165,166], but such activity also alters the normal circadian rhythm of corticosteroid hormone critical in regulating CNS clock control of whole-body physiology as discussed above in Section 2 [167,168], and both perturbations can drive metabolic syndrome [27,155]. Moreover, PVN CRH activity is also a driver of increased SNS tone [169,170,171,172,173,174,175] (adding to the insulin resistance potentiation of hypercortisolemia) that, in turn, can potentiate increases in plasma corticosteroid levels [176]. Furthermore, PVN CRH synthesis and secretion are stimulated by NPY, the synthesis and secretion of which in turn is stimulated by elevated corticosteroid levels, as well as by norepinephrine from the brain stem (see ref. [155] for review of PVN NPY and CRH interactions potentiating elevated SNS tone, increased HPA axis activity, and metabolic syndrome). Additionally, PVN NPY is also a potent stimulus for activation of SNS tone independent of its influence on CRH [177,178]. As such, crisscrossing positive feedback loops between elevated PVN NPY activity and CRH secretion of the obese, insulin-resistant condition initiate and maintain a state of elevated SNS tone and HPA axis overactivity that induces and stabilizes the cardiometabolic syndrome (see Figure 4).

Loss of the circadian peak in dopaminergic input to the SCN couples a transition to both VMH noradrenergic/serotonergic hyperactivity and PVN NPY and CRH hyperactivity to initiate insulin resistance syndrome [27]. This physiological survival response system to low/no food availability (“therapeutic triad”) has transformed into a “treacherous triad” (low SCN circadian peak dopaminergic activity, elevated VMH NE/S activity, and elevated PVN NPY/CRH activity) *when it is chronically, as opposed to seasonally, expressed* that is more than sufficient to initiate and sustain the cardiometabolic syndrome.

## 6. Targeting Low CNS Dopaminergic–Clock Activity to Treat Cardiometabolic Disease: Circadian-Timed Bromocriptine Therapy for the Treatment of Cardiometabolic Syndrome—Preclinical Findings

Since this treacherous triad neuropathology is triggered by the attenuation of CNS dopaminergic activity, particularly by attenuation of the circadian peak of dopaminergic input to the SCN, several studies were conducted to assess the impact of circadian timed dopamine D2 receptor agonist therapy with bromocriptine (to re-establish the natural circadian peak of CNS dopaminergic activity as in the insulin sensitive, glucose tolerant state) upon multiple aspects of cardiometabolic syndrome within peripheral metabolic organs in multiple animal model systems of the disorder. To succinctly summarize these studies in composite, it was found that such circadian-timed bromocriptine treatment in obese, insulin-resistant rodents normalized (attenuated) (a) VMH NE and S input overactivity [138,179] and (b) elevated PVN NPY and CRH levels [155,180] and resulted in attenuation of elevated plasma norepinephrine, hyperinsulinemia, hyperleptinemia, and hypercortisolemia—while phase adjusting the plasma corticosteroid hormone circadian rhythm towards normal [138,179,181]. As expected, such treatment that ameliorated disruption of these neuroendocrine factors was also associated with reductions in (the circadian peak in) basal and insulin-stimulated hepatic lipogenesis [43,77] and simultaneous reductions in several hepatic master transcriptional activators of lipogenesis, gluconeogenesis, and free fatty acid oxidation [179]. Such bromocriptine treatment also produced reductions in body fat stores, insulin resistance, glucose intolerance, hepatic glucose output and whole body FFA oxidation rates [179,181,182], as well as reductions in liver fat content [179,183,184]. Such simultaneous reductions in hepatic lipogenesis and fatty acid oxidation were associated with improved hepatic insulin actions to increase glucose disposal and inhibit glucose output in part via inhibition of several insulin signal transduction blocking proteins [179,182]. Moreover, as it relates to the cardioprotective effects of this therapy, normalization of these hepatic metabolic pathways was coupled to treatment reductions in several liver proinflammatory pathway proteins that contribute to cardiovascular inflammation [179] (see below). Bromocriptine treatment also reduced adipose lipolysis, and this effect, concurrent with its actions on liver metabolism, resulted in a reduction of plasma triglyceride and FFA levels [181]. Reduction in elevated plasma FFA is well-known to reduce insulin resistance; however, it is less well appreciated that elevated plasma FFA feedback centrally inhibits dopamine synthesis and maintains elevated SNS tone, thereby contributing to the maintenance of cardiometabolic syndrome [185,186,187,188,189,190,191,192,193].

Consistent with these effects on liver and adipose metabolism, circadian-timed bromocriptine treatment of seasonally insulin-resistant hamsters also increased glucose disposal during both steady-state euglycemic and hyperglycemic hyperinsulinemic protocols, as well as improved glucose tolerance (reduced AUC glucose and insulin) [181,182]. Similarly, in a study of high fat/fructose-fed rats, bromocriptine treatment improved muscle insulin-stimulated glucose disposal concurrent with a reduction in the pro-inflammatory/insulin signaling blockade IL-6/JAK2/p-STAT3/SOCS3 pathway activity and an increase in the PPAR-γ/adiponectin signaling pathway activity [194]. This improved insulin sensitivity response was also observed in insulin-resistant, high-fat-fed dogs (assessed during a sequential euglycemic and hyperglycemic—hyperinsulinemic clamp) that also was accompanied by improved glucose tolerance and changes in several muscle tissue levels of insulin signal transduction proteins that resultantly potentiate insulin action [195]. Such dog treatment with bromocriptine also improved liver lipid profile, consistent with a reduction in lipogenesis.

It was found that appropriately circadian-timed bromocriptine treatment to improve muscle and liver insulin sensitivity was most pronounced during the postprandial state [196], and further studies indicated that this effect was in part the consequence of such treatment to reverse aberrations in hypothalamic post-meal glucose sensing that potentiate impaired glucose tolerance [148]. In insulin-resistant states, VMH sensing of ambient increases in meal-time-related glucose and FFA is attenuated, resulting in a diminished normal VMH efferent response that would otherwise act to improve post-meal glucose disposal and inhibit both hepatic glucose output and adipose FFA mobilization (reviewed in [148]). This cardiometabolic syndrome aberration in VMH fuel sensing that induces glucose intolerance was reversed by circadian administration of bromocriptine to mimic the natural circadian peak of CNS dopaminergic activity in healthy animals but not by such administration outside this circadian time window [148]. Since postprandial hyperglycemia and hypertriglyceridemia are contributors to CVD, these dopamine–clock interactions to regulate CNS control of postprandial fuel metabolism factor into the cardioprotective effects of bromocriptine-QR in T2D subjects (see discussion below). Fuel substrate disposition studies revealed that circadian-timed bromocriptine therapy shifted energy expenditure away from lipogenesis and towards an increase in protein turnover, resulting in a decrease in the body fat without appreciable lean mass loss (i.e., selective fat loss) [181]. Additionally, while bromocriptine treatment has been observed to reduce SNS tone in the liver, vasculature, white adipose, and immune cells, additional studies demonstrated that bromocriptine treatment actually increased SNS activity at brown fat stores to increase energy expenditure [197]. This finding is concordant with studies wherein exogenous elevation of VMH NE activity in lean insulin-sensitive animals (mimicking VMH responses to *low* CNS dopaminergic tone as described above) resulted in the “whitening” of brown fat [142]. In other investigations, bromocriptine additions directly to white adipose were found to increase FFA oxidation (in addition to its white adipose effects in other studies to reduce lipolysis and FFA mobilization [179,181,182]) to reduce body fat [198]. In agreement with its effect on increasing protein turnover and brown fat energy expenditure, several studies have demonstrated a bromocriptine effect to selectively reduce body fat without majorly altering food consumption [199]. However, despite the beneficial effects of circadian-timed bromocriptine on intermediary peripheral fuel metabolism to reduce hyperinsulinemia, insulin resistance, glucose intolerance, hyperleptinemia, leptin resistance, fatty liver, hyperFFAemia, postprandial hyperglycemia and hypertriglyceridemia, its effect to attenuate the pro-oxidative/pro-inflammatory state in metabolic and immune tissues in animal models of cardiometabolic disease [180] (and human T2D, [200]) may represent the most significant mechanism by which bromocriptine-QR therapy reduced CVD risk in T2D subjects [201,202,203,204].

The neuroendocrine pathophysiological state of concurrent elevated SNS tone and low CNS dopaminergic activity that typifies cardiometabolic syndrome is a strong driver of reactive oxygen species (ROS) and inflammation within the liver, immune, and vascular tissues that ultimately contributes significantly to cardiovascular disease (reviewed in [180,200]). This neuroendocrine proinflammatory effect is both direct upon these liver, immune, and vascular tissues, as well as indirect via induced insulin resistance, hyperinsulinemia, hypercortisolemia (inappropriately elevated plasma corticosteroid during the nadir portion of its daily rhythm), leptin resistance, hyperleptinemia, and elevated plasma prolactin (inappropriately elevated plasma prolactin during the nadir portion of its daily rhythm). These pathophysiological consequences have been observed to be ameliorated by circadian-timed sympatholytic, dopamine agonist bromocriptine therapy in various animal models of cardiometabolic syndrome [180,194,195,196,197,198,199]. Significantly, bromocriptine is also known to reduce pro-inflammatory immune reactions in a variety of non-metabolic-induced animal models of inflammation and in certain human autoimmune disease states (reviewed in [200]).

There exists a potent self-amplifying positive feedback loop between pro-oxidative state generation within liver, immune, and vascular cells (facilitated by the treacherous triad) and proinflammatory state within these tissues that culminates in vascular tissue endothelial nitric oxide synthase (eNOS) uncoupling, an oxidative stress response that causes eNOS to generate pleiotropic cardiovascular damaging reactive oxygen/nitrogen species (ROS/RNS) instead of pleiotropic cardiovascular protective nitric oxide leading to endothelial dysfunction driven vascular disease (vasoconstriction, vascular smooth muscle cell proliferation, vascular inflammation, immunocyte vascular infiltration, atherosclerosis, arteriosclerosis, vascular cell death, and cardiovascular remodeling, as well as direct myocardial tissue damage) (see refs. [180,200] for thorough reviews). If left unabated, this positive feedback loop can inflict major damage to the vasculature not reliant on any change in plasma lipid or cholesterol profile [180,200].

As described above, treatment of hypertensive SHR rats with the sympatholytic dopamine D2 receptor agonist, bromocriptine, that corrected (attenuated) the elevated VMH NE and S activities and ameliorated pathological biochemistry of fatty liver also reduced the hepatic levels of several transcription factors (NFkB, IKKαβ, SOCS3, JNK) for transcription of multiple proinflammatory proteins that are secreted into the circulation and promote vascular inflammation [179]. Additionally, as described above, bromocriptine treatment of high fat-fed rodents reduced proinflammatory factors in muscle while improving insulin sensitivity [194]. Moreover, circadian-timed bromocriptine treatment of hypertensive insulin-resistant SHR rats held on a high-fat diet reduced aortic oxidative stress and re-coupled uncoupled eNOS to increase functional eNOS activity that resultantly reduces endothelial dysfunction and vascular damage [180]. Such bromocriptine treatment also reduced systolic and diastolic blood pressure in these animals, an effect consistent with its sympatholytic, dopaminergic activity, well described in the literature. This bromocriptine-induced improvement (attenuation) of vascular pro-oxidative/pro-inflammatory status (and endothelial dysfunction) is in good agreement with another study wherein bromocriptine treatment of high fructose-fed rats led to improvement of aortic endothelial relaxant response to acetylcholine and reduced response to the pressor effects of norepinephrine, epinephrine, and phenylephrine and protection from cardiac hypertrophy and myocardium degeneration [205]. Finally, bromocriptine has been observed to reduce oxidative stress/inflammation associated with ischemic/reperfusion injury and apoptosis of cardiomyocytes and isolated heart, as well as of the kidney and also to protect against ischemia-induced hippocampal neurodegeneration [206,207,208,209,210].

In summary, the composite of animal studies on CNS dopamine regulation of cardiometabolic physiology indicates that the phase and amplitude of the circadian rhythm of CNS dopaminergic input activity to the SCN area/clock circuitry critically regulate its output control of metabolic and immune functions that modulate cardiometabolic physiology. The natural seasonal diminution of the circadian peak in dopaminergic input activity to the SCN clock circuitry among animals in the wild or held under natural conditions in the laboratory programs the clock output functions to chronically elevate SNS tone and the HPA axis and thereby potentiate insulin resistance and a pro-inflammatory state. While this evolutionarily preserved seasonal mechanism to self-initiate the insulin-resistant condition acts to sustain survival against stresses of seasonal low/no food availability in the wild, its persistent (years long) expression is detrimental to cardiometabolic health. Moreover, chronically decreased CNS dopaminergic tone has been observed in a variety of genetic or induced animal models of cardiometabolic disease, including ob/ob mice, db/db mice, A^Y^/a agouti mice, high fat-fed rodents, as well as hypertensive SHR rats (reviewed in [27]) and (circadian-timed) dopamine agonist treatment to reverse this diminution in CNS circadian peak dopaminergic activity improved several metabolic aspects of the condition in ob/ob mice, db/db mice, A^Y^/a agouti mice, Zucker rats, high fat-fed rodents, hypertensive SHR rats, and early weaned rodent pups at adulthood, obese pigs, and high fat fed dogs [179,180,183,195,199,211,212,213,214,215,216]. In general, this treatment method ameliorated several metabolic derangements, including hyperinsulinemia, insulin resistance, glucose intolerance, hyperleptinemia, leptin resistance, fatty liver, hyperFFAemia, obesity, and postprandial hyperglycemia and hypertriglyceridemia. Additionally, and importantly, in cardiometabolic disease animal models, this bromocriptine treatment attenuated (a) the liver and vascular pro-oxidative/pro-inflammatory state and (b) vascular endothelial dysfunction and subsequent cardiovascular disease. What is the evidence that alterations in brain dopamine–clock interactions are present and operative in the development of human cardiometabolic disease and that the disorder can be improved by appropriately circadian-timed bromocriptine therapy? This question must be addressed first by a discussion of documented circadian biology in humans generally and, more specifically, as it relates to cardiometabolic physiology.

## 7. Circadian Rhythm Influence on Human Physiology

Not unexpectedly, circadian rhythms of a large number of neural and endocrine activities have been documented in humans to date as in other studied mammalian species [130,217,218,219,220,221,222,223,224,225,226,227,228,229,230,231]. Since the initial demonstrations of circadian responses to hormones and the critical role of temporal synergisms of circadian rhythms of neuroendocrine activities in the manifestation of the annual cycle in vertebrates almost 60 years ago [35,39], the field of circadian science research has exploded. A recent search of the National Institute of Health, National Library of Medicine, and PubMed term “circadian” with/without “human” filter yielded 61,312/103,018 hits, respectively, while the PubMed term “seasonal” with/without “human” filter yielded 119,161/253,317 hits, respectively. As it relates to the present cardiometabolic physiology discussion, the preponderance of evidence clearly indicates that there exist circadian rhythms or daily variations of multiple metabolic control hormones, including prolactin, cortisol, leptin, thyroid stimulating hormone and others in humans as in other vertebrates [27,130,217,218,219,220,221,222,223,224,225,226,227,228,229,230,231]. There are also well-documented circadian rhythms of responsiveness to neuroendocrine control, including insulin sensitivity, adipose lipolysis, hepatic triglyceride synthesis, plasma triglyceride and free fatty acid level in humans [224,225,226,227,228,229,230,231,232,233,234,235,236], thus providing a framework for temporal interactions of stimulus and response rhythms to generate a gradation of target tissue metabolic effects depending upon the phase relationship of the stimulus and response rhythms as is well documented in non-human vertebrate species described in the earlier discussion above (see Section 2, Section 3, Section 4 and Section 5).

Human circadian rhythms of immunity elicit circadian peaks of immune function that potentiate local inflammation towards and attack of pathogens as a function of the interactive phase relationships of numerous cellular stimulus and response rhythms among the many immunocyte types that comprise the immune system [119,120,121,122,231]. Within the cardiovascular system, there are prominent daily rhythms of immunocyte vascular infiltration and proinflammatory activity, ROS generation, platelet aggregation, endothelial function, and blood pressure regulation [237,238,239,240,241,242]. The heart also exhibits circadian rhythms of metabolic functions governing its activity [243,244,245,246]. The interaction of the phase and amplitude of these various circadian activities within the cardiovascular system sets the appropriate daily growth, repair, hemodynamics, and metabolism of its cells/tissues and, thus, the status of cardiovascular health (or disease) [10,245,247,248,249,250,251,252,253,254,255,256,257,258,259,260,261,262,263,264]. As a simple example of postulated pathophysiology emanating from disruption of this circadian interaction, vascular damage is greatest when circadian peaks of cellular/tissue pro-oxidative activities increase and broaden *and temporally coincide* with such altered (increased and broadened) circadian peaks in pro-inflammatory and vasoconstrictive biological functions—resulting in a maximized prooxidative stimulation of a proinflammatory state. Recall that the phase and amplitude of many of these peripheral circadian rhythms are set by the central circadian pacemaker circuit output to the periphery via the neuroendocrine axis. In turn, the temporal relations of circadian multiple neural activities within the central circadian pacemaker circuit are adjusted by internal input from other brain centers and the periphery, as well as from the external environment.

## 8. Shift in CNS Circadian Dopamine–Clock Interactions to Initiate the CNS Treacherous Triad Are Coupled to Known Peripheral Neuroendocrine Pathologies That Potentiate Downstream Cardiovascular Disease in Humans

As reviewed above in Section 4, Section 5 and Section 6, preclinical studies indicate that *seasonal* attenuation of the CNS circadian peak in dopaminergic activity subsequently induces the CNS “therapeutic triad” (decreased dopaminergic input to the SCN, elevated noradrenergic/serotonergic input to the VMH, and elevated NPY input to and CRH release from the PVN) that in turn initiates *and maintains* the CNS hypo-dopaminergic, elevated SNS, elevated HPA axis, insulin-resistant state as a survival response to *seasonal* low food availability stress. In this regard, it is extremely important to appreciate that circannual cycles of metabolism, immunity, behavior, and cardiovascular physiology have been well documented in humans. Under normal circumstances, the physiology of healthy (and metabolic syndrome type) individuals in various locales around the globe oscillates seasonally between a lean, insulin sensitive, reduced systemic inflammatory, normo-coagulative, normotensive state to a (more metabolic syndrome-like) increased body fat store (not necessarily overweight), less insulin sensitive, relatively more pro-inflammatory, pro-coagulative, hypertensive state (that generally coincides with the season of reduced food supply in the wild [e.g., Winter]) [265,266,267,268,269,270,271,272,273,274,275,276,277,278,279,280,281,282,283,284,285]. Furthermore, actual adverse CV events are reported most frequently during this metabolic syndrome-like season [286,287,288,289,290]. *Is there any evidence that seasonal shifts in CNS dopamine–clock interactions driving the “therapeutic triad” that potentiate seasonal insulin resistance for survival in animals under natural conditions are somehow operative long term (years long) in humans to similarly express the chronic metabolic syndrome (thus converting the “therapeutic” triad into the “treacherous triad”) leading to cardiovascular disease?* While it is not currently possible to directly assess in humans such CNS circadian changes in neural (hypothalamic) physiology expressing this treacherous triad, there are several indirect measures of such CNS activities that can and have been made that do strongly suggest that such altered CNS dopaminergic input to the clock circuitry exists in human metabolic syndrome and they may be summarized as follows.

Firstly, a cardiometabolic disease resulting from a diminution of the circadian peak of dopaminergic input activity to the SCN is coupled to such decreases in several brain regions, such as the striatum (dopaminergic input to which is modulated by control from the supramammillary nucleus, the same center regulating dopaminergic input control to the SCN) in study animals [291,292] Several studies (though not all [likely due to time of day variation in measurement]) of obese, insulin-resistant humans have also documented a decrease In striatal dopaminergic activity (either as a consequence of decreased neurotransmitter release or of decreased receptor number or binding affinity) relative to lean, insulin-sensitive counterparts [293,294,295,296,297]. Additionally, administration of alpha-methyl-para-tyrosine, a false dopamine neurotransmitter that results in short-term decreased brain dopamine synthesis, to young, healthy lean insulin-sensitive individuals in a manner that attenuates the normal morning rise in brain dopamine activity induces insulin resistance within only a day or two [298,299]. Similarly, administration of the dopamine D2 receptor agonist, bromocriptine, to obese, insulin-resistant individuals at the time of the daily peak in CNS circadian dopaminergic activity (near waking time in the morning) but not when administered in the evening improved postprandial insulin sensitivity [300]. Secondly, seasonal shifts in brain dopamine and serotonin neurochemistry, an occurrence associated with seasonal physiological shifts in animals, have been observed in healthy humans [275,301], and attenuation of brain dopamine activity, a major regulator of serotonin functions [302,303], can allow for shifts in circadian serotonergic activity. Peripheral expressions of CNS dopamine and serotonin circadian activities in animals include the circadian rhythms of plasma prolactin and cortisol, respectively, whose phase relationship regulates the expression of the lean, insulin-sensitive or obese, insulin-resistant state in large part by feeding back centrally to set the CNS dopamine and serotonin activity rhythms as described above in Section 4. In fact, the phase relationship of the circadian rhythms of plasma prolactin and cortisol likewise differ between obese, insulin resistant and lean, insulin-sensitive humans [304] as in studied animals across the vertebrate subphylum described above in Section 3 and Section 4. Thirdly, an increase in PVN NPY and CRH activities observed in cardiometabolic syndrome rodents are strong stimuli leading to increases in SNS tone and HPA axis activity and such (often concurrent) neuroendocrine aberrations are often observed in human cardiometabolic syndrome [305,306,307,308,309,310,311,312,313,314,315,316,317,318,319,320,321,322]. Lastly, increases in noradrenergic/serotonergic input activities to the VMH observed in animal models of cardiometabolic syndrome drive increases in SNS tone and leptin resistance that induce postprandial insulin resistance and glucose intolerance, hypertension, and obesity, all common features of human cardiometabolic syndrome [323,324,325,326,327,328,329,330,331,332,333,334,335,336,337,338,339,340,341,342]. *The very critical point here is that elevations in SNS tone (with or without increased HPA axis activity) to the liver, white adipose, muscle, vasculature, heart, and immune system not only merely associate with but drive multiple aspects of the cardiometabolic syndrome* [305,306,307,308,309,310,311,312,313,314,315,316,317,318,319,320,321,323,324,325,326,327,328,329,330,331,332,333,334,335,336,337,338,339,340,341]. In total, both naturally evolved seasonal metabolic syndrome-like physiology and pathological chronic cardiometabolic syndrome are generally typified by decreased CNS dopaminergic activity, increased SNS tone to white adipose, liver, muscle, and the cardiovascular system (with a possible decrease to brown fat [197]), increased central and peripheral leptin resistance, and increased HPA axis activity. The composite of this neuroendocrine shift potentiates the cardiometabolic syndrome. *What environmental (or genetic) factors may predispose for the inappropriate amplification and prolongation of the natural (seasonal) circadian neuroendocrine induction of the “therapeutic triad” to manifest its years-long sustained expression (the “treacherous triad”) precipitating cardiometabolic disease and associated CVD observed in humans?* Such factors would necessarily attenuate brain dopamine activity and alter brain clock circuit functions regulating the autonomic and endocrine control of peripheral fuel metabolism and cardiovascular biology.

## 9. Common Chronic Environmental Stress Factors of Modern Man Disrupt the CNS Dopaminergic–Clock Circuit, Reduce CNS Dopaminergic Activity and Increase SNS Tone to Potentiate Cardiometabolic Disease and CVD

**Figure 5:**
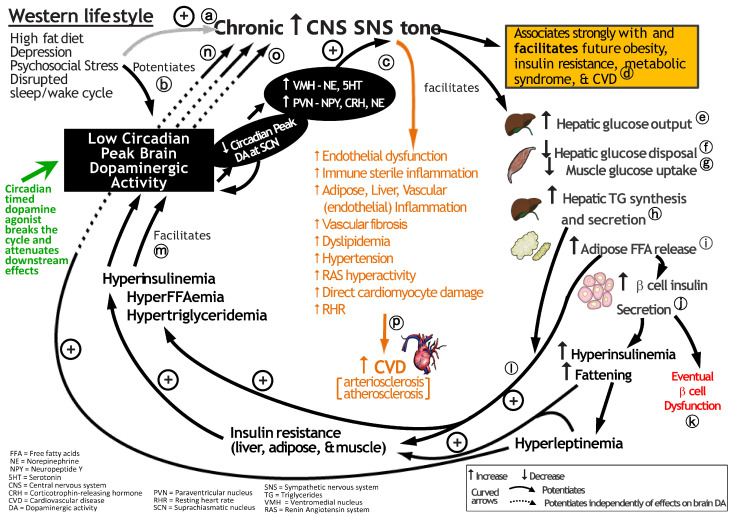
CNS hypodopaminergic activity alters CNS clock output to induce chronic elevated SNS tone that initiates a positive feedback loop to maintain the CNS hypodopaminergic/elevated SNS tone expression and precipitate cardiometabolic disease. Decreased CNS circadian dopaminergic activity, induced by Western lifestyle “stressors”, encompasses decreased circadian peak dopaminergic activity at the pacemaker clock (SCN) that effectuates an increased CNS SNS tone which collectively potentiates metabolic syndrome. Biochemical aspects of the metabolic syndrome then positively feed back centrally via multiple pathways to maintain this neuropathological state. Within the cycling events of this positive feedback system are sustained systemic pro-oxidative/pro-inflammatory activities that potentiate cardiovascular disease. References for Figure 5: ⓐ [94,188,367,368,369,370,371,372,373]; ⓑ [186,187,293,294,295,349,374,375,376,377,378,379]; ⓒ [141,142,149,155,179,380,381]; ⓓ [312,313,340,382,383,384,385,386,387,388,389,390,391,392], ⓔ [305,393,394,395,396,397,398], ⓕ [394,395]; ⓖ [305,399,400]; ⓗ [401,402,403]; ⓘ [403,404]; ⓙ [141,142,381]; ⓚ [405]; ⓛ [188,406,407,408,409,410,411,412]; ⓜ [94,185,188,189,297,344,345,346,375,413,414,415,416,417,418,419]; ⓝ [420,421,422,423,424,425]; ⓞ [426,427]; ⓟ [179,392,428,429,430,431,432,433,434,435,436,437,438,439,440,441,442].

Chronic environmental stressors in humans, including Western diets (high saturated fat/simple sugar [including in particular high fructose] diets), altered sleep/wake architecture (photoperiod disruptions including shift work, shortened or prolonged sleep cycle duration, sleep apnea, interrupted sleep, light exposure during sleep, insomnia), or psychosocial stress and depression, are known to alter CNS clock function, attenuate CNS dopaminergic activity and also elevate SNS tone (and the HPA axis) (i.e., manifestations of the hypothalamic treacherous triad) and concurrently associate with cardiometabolic syndrome and CVD [4,5,6,7,8,9,10,11,12,13,14,15,16,17,18,19,20,49,185,297,324,341,343,344,345,346,347,348,349,350,351,352,353,354,355,356,357,358,359,360,361,362,363,364,365,366]. The common denominator of the CNS-altered clock—hypodopaminergic/elevated SNS tone (overactivated HPA axis) neuroendocrine state (neuroendocrine output of the treacherous triad)—among these various stressors’ pathway towards cardiometabolic disease can hardly be overstated. Such findings suggest that these chronically expressed aberrant environmental cues are processed by the CNS dopaminergic input to the clock system as a stress signal prompting a clock-driven neuronal induction of a metabolic survival response—the therapeutic triad-induced insulin-resistant state to maintain a glucose supply to the CNS. However, if sustained long term, this CNS-clock response system becomes the treacherous triad that sets up a positive feedback loop between itself and the peripheral neurometabolic state it creates, and chronic cardiometabolic disease is potentiated (see Figure 5) as demonstrated in numerous preclinical studies described above in Section 6.

**Figure 6:**
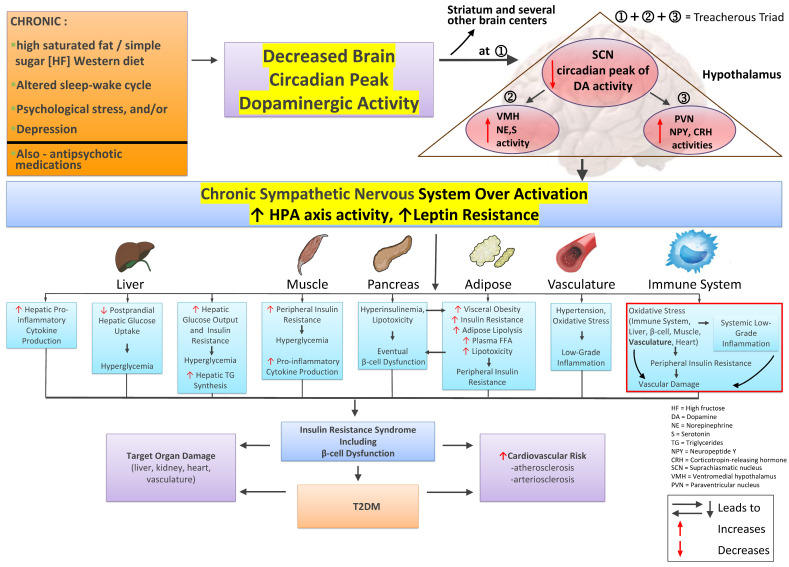
Evidence-based proposed mechanisms of CNS clock-alterations that drive metabolic syndrome: Western lifestyle perturbations to normal physiology (chronic high fat/simple sugar diets, altered sleep/wake architecture, psychosocial stress and/or stimulated depression) potentiate a reduction in CNS circadian peak dopaminergic activity at the pacemaker clock to initiate the hypothalamic treacherous triad leading to neuroendocrine shifts driving metabolic syndrome and its sequelae.

Such *chronic* environmental stressors that shift the CNS clock circuit temporal organization to amplify *and lock* the individual in the seasonal insulin-resistant neurometabolic physiology for years on end can lead to cardiometabolic and cardiovascular disease (see Figure 6).

Importantly, as it relates to cardiovascular disease specifically, all of the above listed environmental stress factors (high saturated fat/high simple sugar diet, altered sleep/wake architecture [photoperiod disruptions including shift work, shortened or prolonged sleep cycle duration, sleep apnea, interrupted sleep, light exposure during sleep, insomnia], or psychosocial stress and depression) depress brain dopamine activity and alter brain clock circuitry to initiate the treacherous triad (chronic low brain dopaminergic and increased SNS [and increased HPA axis] activities) which in turn acts as a strong stimulus for systemic low-grade inflammation [17,18,20,64,200,259,260,261,262,263,264,293,294,311,347,348,356,359,443,444,445,446], a major driving force for CVD [17,18,20,200,447,448,449]. Of note, high fructose (and sucrose) containing diets not only reduce brain dopaminergic activity (contributing to the attenuation of satiety and initiation of the treacherous triad) [450,451,452] but also directly stimulate hepatic de novo lipogenesis, fatty liver, and increased (atherogenic) postprandial triglyceride-rich lipoprotein levels [22,23,24,25]. Since hepatic de novo lipogenesis and triglyceride secretion can be potentiated by the treacherous triad, consumption of high levels of fructose (or sucrose) can offer a particularly potent double negative hit on both increased fat stores and CVD [22,23,24,25]. Respecting the impact of dietary saturated fat on CVD, the relationship is more nuanced. *Chronically* increased dietary saturated fat consumption is known to inhibit CNS dopaminergic activity in animals and humans, as described above, yet clinical studies of reducing dietary saturated fat have not demonstrated overwhelming evidence of reduced CVD outcomes [6,7]. However, there are few, if any, long-term randomized controlled clinical trials assessing the impact of dietary saturated fat on CVD outcomes wherein both on-study accompanying nutrient mix (fats, carbohydrates, simple sugar types) and baseline metabolic status (e.g., degree of obesity and insulin resistance) were each controlled for. Significant evidence indicates that replacing dietary saturated fats with dietary n-3 or n-6 polyunsaturated fats and/or Mediterranean based fat (mixed mono and poly-unsaturated fats) does reduce CVD event rate [reviewed in [6,7], *and importantly such replacement stimulates a return to normal CNS dopaminergic function* [186,453]. Moreover, increased levels of stored saturated fatty acids released from adipose into the circulation induce feedback centrally to induce the treacherous triad and also stimulate cellular and tissue cardiometabolic pathology that are each reversed by oleic acid replacement or addition in multiple animal studies [454,455,456]. The “western diet”, which is a composite of high saturated fat and simple sugar [e.g., high fructose], offers a potent and pleiotropic stimulus for hypothalamic treacherous triad induction and may potentiate CVD risk over the long term (e.g., years in humans).

The plethora of data from multiple angles of investigation among several different laboratories reviewed herein thus far clearly indicate an important role for CNS circadian dopaminergic communication with the brain clock circuit in the control of whole-body fuel metabolism and cardiovascular health. Results of clinical studies investigating the effect of circadian-timed bromocriptine-QR therapy to improve cardiometabolic syndrome and T2D in humans largely reflect the findings observed in similar preclinical studies across multiple animal model systems of insulin resistance described above as detailed below.

## 10. Circadian-Timed Bromocriptine-QR for the Treatment of Cardiometabolic Disease–Human Studies

Bromocriptine-QR is a unique formulation of specifically micronized bromocriptine mesylate that provides for rapid dissolution and absorption from the gut with a relatively short half-life, thus generating a brief pulse of bromocriptine to the circulation [457] and by extension to the CNS [458,459]. Early morning (within 2 h of waking) circadian-timed administration of bromocriptine-QR is intended to mimic the early morning circadian peak in central dopaminergic activity observed in healthy non-T2D subjects, diminished expression of which in insulin-resistant states contributes to the development of the condition [298,299,457] as demonstrated in numerous animal models of insulin resistance as described above in Section 2, Section 3, Section 4, Section 5 and Section 6.

**Figure 7:**
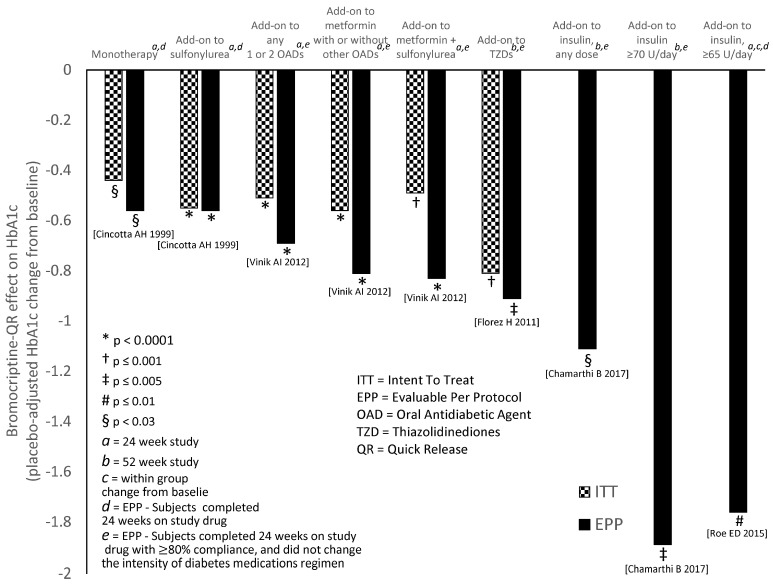
Circadian-timed Bromocriptine-QR therapy improves glycemic control versus placebo as an add-on therapy to a variety of anti-diabetes agents. References for Figure 7: Cincotta AH 1999 [460]; Vinik AI 2012 [463]; Florez H 2011 [464]; Chamarthi B 2017 [466]; Roe ED 2015 [465].

In pre-diabetes subjects, as in preclinical studies of metabolic syndrome animals, such circadian-timed (morning within 2 h of waking) bromocriptine-QR therapy has been observed to reduce postprandial hyperglycemia and hyperinsulinemia across the standard meals of the day (breakfast, lunch, and dinner) [380]. Similarly, in T2D subjects, such bromocriptine-QR therapy reduced postprandial hyperglycemia, without raising plasma insulin levels, across the standard meals of the day [460]. Bromocriptine-QR therapy of T2D subjects was also demonstrated to increase maximally stimulated insulin-mediated glucose disposal assessed in the fasting state via euglycemic hyperinsulinemic clamp methodology [461]. Assessment of bromocriptine-QR therapy upon whole-body glucose balance during the postprandial state (via radiolabeled glucose ingestion at the meal) in T2D subjects whose glycemia was inadequately controlled on GLP-1 receptor agonist revealed that the treatment reduced glucose rate of appearance and postprandial glucose level, without raising the plasma insulin level, presumably by increasing hepatic glucose extraction (the treatment has no known effect to inhibit gastrointestinal absorption of glucose and its parenteral administration in a variety of animal studies described above attenuated the insulin-resistant syndrome) and also by reducing hepatic glucose output (i.e., attenuating two major liver glucose metabolism abnormalities in T2D) [462]. The predominant beneficial effect of bromocriptine-QR on glycemic control in prediabetes and T2D is upon postprandial hyperglycemia, consistent with its effect to improve VMH glucose sensing of local hyperglycemia (meal-associated levels) [148], which in turn activates post-meal peripheral glucose disposal mechanisms [146,147]. The effects of bromocriptine-QR on HbA1c across various study populations vary from 0.5 to 1.8 reduction versus placebo [460,463,464] (Figure 7), depending upon the concomitant anti-diabetes medication (including insulin [465,466]) and/or level of elevated SNS tone-associated biomarkers (elevated resting heart rate, hypertension with elevated plasma triglyceride levels) at baseline [380,457,465,466,467,468].

In general, the impact of circadian-timed bromocriptine-QR therapy to reduce HbA1c in T2D subjects increases as the baseline post-meal plasma insulin level of the subject increases [380], in agreement with its effect to improve post-meal insulin-mediated glucose disposal. The therapy is particularly effective in subjects on basal/mealtime insulin (with metformin), again likely owing to its insulin-sensitizing effects (HbA1c reductions of 1.10–1.89 vs. placebo over 52 weeks) [465,466]. Additionally, in T2D subjects with signs of particularly elevated SNS tone, such as elevated resting heart rate (≥80 beats per minute) or presence of hypertension plus hypertriglyceridemia, the therapy has been noted to produce HbA1c reductions of 1.2–1.3 versus placebo, while also reducing elevated resting heart rate (by 4–10 beats per minute) or plasma hypertriglyceridemia and hypertension, respectively [457,467,468]. As the levels of these biomarkers associated with elevated SNS tone increase, so too does the magnitude of the impact of circadian-timed bromocriptine-QR to reduce HbA1c level. These clinical findings are in very good agreement with those of similar preclinical studies of time-of-day dependent CNS dopamine or systemic bromocriptine effects on metabolism, as detailed above in Section 6. Most importantly, in clinical studies assessing the time-of-day-dependent effects of bromocriptine to improve post-glucose load insulin resistance in obese individuals, the effect was present when administered at the onset of waking (time of day of natural peak CNS dopaminergic activity in healthy insulin-sensitive individuals) but not when administered outside of its natural CNS peak activity late in the day [300] as observed in preclinical studies (see Section 6).

**Figure 8:**
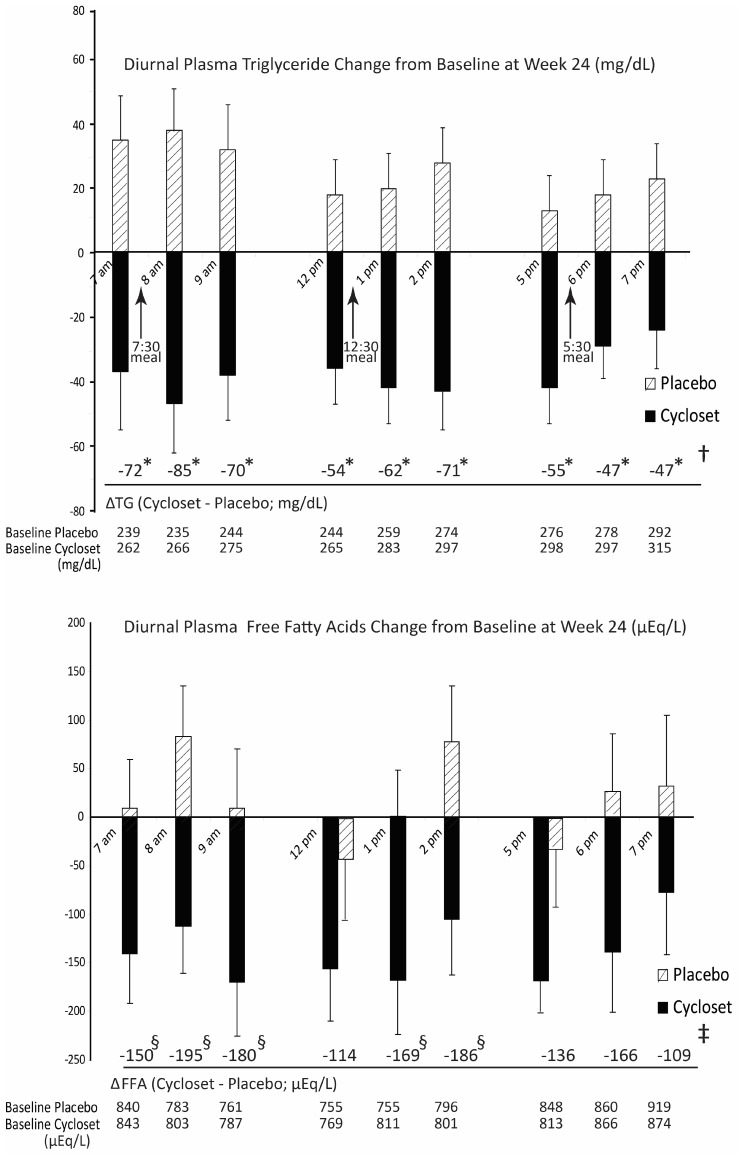
Circadian-timed Bromocriptine-QR therapy reduced day-long (across the three standard meals of the day) plasma triglyceride and free fatty acid levels versus placebo in T2D subjects whose glycemia was inadequately controlled on sulfonylurea. Updated from [460,469]; Copyright 1999 Ashley Publications Ltd. – current: Informa UK Ltd. From Expert Opin Investig Drugs^®^ Vol. 8, 1999; 1683-1707 [460], updated with permission. Copyright 2010 Taylor and Francis, www.tandfonline.com. From Expert Opin Pharmacother^®^ Vol. 11, 2010; 269-279 [469], updated with permission. * Denotes a significant difference from placebo (*p* < 0.005). § Denotes a significant difference from placebo (*p* < 0.05). † A two-way repeated measures ANOVA on treatment and hour with interaction demonstrated a significant treatment effect over the entire day (*p* < 0.0001). ‡ A two-way repeated measures ANOVA on treatment and hour with interaction demonstrated a significant treatment effect over the entire day (*p* < 0.02). X axis represents time of day between 7 am and 7 pm.

Circadian-timed bromocriptine-QR therapy has also been demonstrated to reduce elevated levels of plasma triglyceride (29%) and FFA (19%) across the diurnal portion of the day (including meal times) in T2D subjects [460], as in animal studies (see Section 6) (Figure 8).

**Figure 9:**
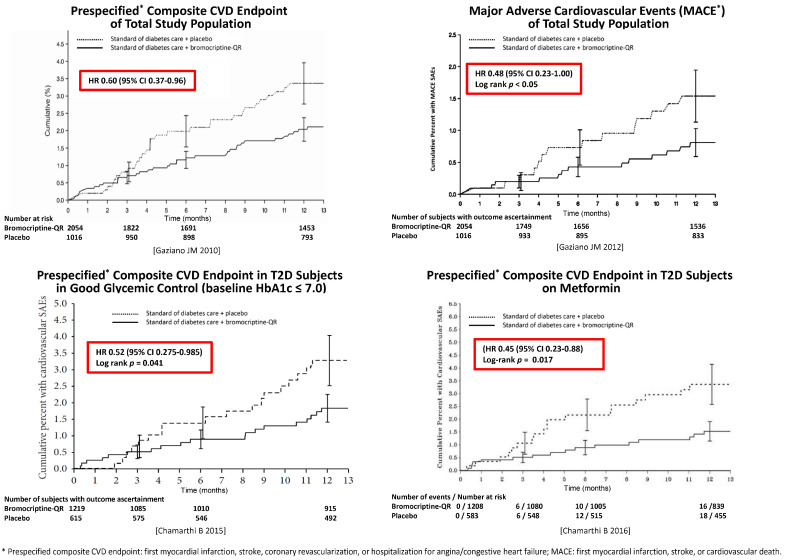
Circadian-timed Bromocriptine-QR therapy was associated with reduced cardiovascular event rate versus placebo in T2D subjects in multiple analyses of study groups from the Cycloset Safety Trial. Gaziano JM 2010 [201], Copyright 2010 American Diabetes Association. From Diabetes Care.®, Vol. 33, 2010; 1503-1508 [201], updated with permission; Gaziano JM 2012 [202], Copyright 2012 The Authors; Chamarthi B 2015 [203], Copyright 2012 The Authors; Chamarthi B 2016 [204], Copyright 2016, Taylor and Francis, www.tandfonline.com. From Postgrad Med® Vol. 128, 2016; 761-769 [204], updated with permission.

Owing to its sympatholytic effects [458,470], bromocriptine therapy has long been demonstrated to reduce hypertension [471], and the effects of Cycloset to reduce elevated resting heart rate are coupled with reductions in systolic and diastolic blood pressures (−3.6 and −1.9 mmHg relative to placebo, respectively), an indicator of reduced sympathetic tone [457]. While bromocriptine-QR therapy effects to simultaneously reduce insulin resistance, postprandial hyperglycemia and dyslipidemia, elevated resting heart rate, and hypertension can certainly contribute to a reduced cardiovascular disease risk, this composite effect alone simply cannot explain the significant rapid (within 1 year) and robust (40–55%) reduction of adverse cardiovascular events observed with the therapy in the Cycloset Safety Trial (CST) of T2D subjects [201,202,203,204]. Among several different analyses of adverse cardiovascular outcomes in the one-year CST, circadian-timed, bromocriptine-QR therapy was found to significantly reduce the composite cardiovascular (CV) endpoint of myocardial infarction, stroke, and hospitalization for unstable angina, revascularization surgery, and heart failure (with or without CVD death included), as well as of the composite CV endpoint of myocardial infarction, stroke, and CV death by between 40–55%. This bromocriptine-QR effect was demonstrable even among T2D subjects in good glycemic control at baseline (HbA1c ≤ 7.0) [203]. Moreover, the study subjects from the CST were largely (>75%) without established preexisting cardiovascular disease [201,202,203,204] (Figure 9).

As observed preclinically, a growing body of clinical evidence implicates circadian-timed bromocriptine-QR therapy attenuation of the immune pro-oxidative/pro-inflammatory state and improvement of endothelial dysfunction that is each typical of and dominant in the development of the cardiometabolic syndrome and CVD as major mechanisms of the therapy’s beneficial impact on CVD [200,462].

Endothelial dysfunction is generally recognized as the initial insult to the vasculature that leads to CVD [472,473,474,475,476]. Endothelial dysfunction in cardiometabolic syndrome is largely a consequence of a biochemical phenomenon termed endothelial nitric oxide synthase (eNOS) uncoupling [477,478,479,480]. Uncoupling of the eNOS enzyme dimer prevents its function from synthesizing nitric oxide (NO) from the arginine substrate. NO is a major autocrine/paracrine stimulus for the maintenance of vascular health (stimulation of processes responsible for vasodilation and endothelial cell repair and health and inhibition of local inflammation, immunocyte infiltration, smooth muscle cell proliferation, adhesion molecule aggregation, fibrosis, and arteriosclerosis). Moreover, eNOS uncoupling not only reduces NO production but also alters the function of the enzyme to now generate a variety of reactive oxygen and reactive nitrogen species (ROS and RNS, respectively) that, via pleiotropic mechanisms, induce vascular inflammation, immunocyte vascular infiltration, arteriosclerosis and atherosclerosis. Worse yet, since ROS and RNS produced by uncoupled eNOS feedback to stimulate further eNOS uncoupling and eNOS transcription, a vascular destructive self-sustaining cyclical loop is generated [180,200,477,478,479,480]. Increased SNS tone in conjunction with a hypodopaminergic state also acts upon the circulating immunocytes to stimulate a pro-oxidative/pro-inflammatory state leading to serious damage to the vasculature via immunocyte infiltration and extracellular matrix expansion, vascular cell proliferation and calcification and further eNOS uncoupling [180,200]. Additionally, chronically elevated SNS tone in conjunction with a hypodopaminergic state stimulates the induction of insulin resistance, hypertriglyceridemia, and hypertension—all of which also drive vascular eNOS uncoupling and immune-inflammatory damage [180,200,331,428,481,482]. As such, chronically elevated SNS tone/CNS hypodopaminergic state is a pleiotropic stimulus that initiates and sustains vascular eNOS uncoupling and inflammation/damage, thus potentiating CVD. Preclinical studies with circadian timed bromocriptine indicate that the treatment reversed elevated SNS tone and biochemical processes of metabolic syndrome, liver inflammation, and vascular eNOS uncoupling in hypertensive, metabolic syndrome SHR rats [179,180]. Importantly, bromocriptine-QR therapy of human T2D subjects resulted in an improvement in endothelial dysfunction and hypertension [462].

These bromocriptine-QR clinical study findings regarding its attenuation of endothelial dysfunction led us to investigate in these same T2D subjects the impact of the therapy upon peripheral blood mononuclear cell pro-oxidative/pro-inflammatory status, elevations of which are a major driver of endothelial dysfunction, vascular inflammation, and CVD. It was found that such circadian-timed bromocriptine-QR therapy in T2D subjects reduced a wide range of peripheral blood mononuclear cell (PBMC) gene expressions (25 different genes) for proteins involved in endoplasmic reticulum stress, whole-cell oxidative stress, response to existing oxidative stress, and activation of potent Toll-like receptor (TLR) receptor pathways for inflammatory cytokine production and adhesion molecule synthesis and secretion. Each of PBMC (and endothelial cell) endoplasmic reticulum stress, whole-cell oxidative stress, and TLR proinflammatory pathways activation is known to contribute to cardiometabolic disease risk, particularly arterial stiffness (reviewed in [200]). These bromocriptine-QR-induced reductions in multiple pro-oxidative/pro-inflammatory pathway gene expressions within the PBMCs were coupled to reductions in plasma pro-inflammatory cytokines, proteins and markers of oxidative stress, as well as of norepinephrine and normetanephrine (a measure of SNS tone) and endothelial dysfunction [200,462]. Although the breadth and magnitude of these results may be notable, the direction of response should not be so surprising since each chronically elevated sympathetic tone and CNS hypo-dopaminergic activity have been demonstrated to potentiate immune sterile inflammation and systemic oxidative stress (reviewed in [200]), and each of these neuroendocrine aberrations has been observed to be ameliorated by circadian-timed bromocriptine-QR therapy in preclinical and clinical studies (as described and referenced herein). In addition to this pathophysiological neuroendocrine effect of chronically elevated sympathetic tone and CNS hypo-dopaminergic activity on the immune-vascular axis, it should be recognized that the CNS hypodopaminergic activity/elevated SNS tone—to the liver potentiates hepatic inflammatory cytokine secretion, triglyceride synthesis and secretion, and insulin resistance [179,332,401,402,483,484]—to the adipose, potentiates inflammation and FFA secretion that induces systemic insulin resistance and vascular damage [485,486,487,488]—to the kidney, stimulates the renin-angiotensin system that potentiates vascular damage [489,490,491,492,493,494,495,496],—to the heart, stimulates cardiomyocyte inflammation and damage and heart failure [200,262,263,497,498,499,500,501,502,503,504,505]—that all have been observed to be ameliorated by bromocriptine as mentioned above. Consequently, since circadian-timed bromocriptine-QR therapy of T2D/metabolic syndrome simultaneously reverses both the elevated SNS tone and CNS hypo-dopaminergic activity to directly attenuate this immune/vascular pro-oxidative/pro-inflammatory state, as well as systemic dysmetabolism that adds to this immune/vascular pathology, it should not be unexpected that the therapy produces a significant (and rather rapid) reduction in CV events in T2D subjects [201,202,203,204]. In good agreement with such bromocriptine-QR cardiovascular findings in T2D subjects are the recent findings that such bromocriptine-QR therapy of T1DM subjects resulted in rapid (within 4 weeks) reductions in arterial stiffness and blood pressure [506]. Moreover, bromocriptine has been observed to significantly reduce postpartum cardiomyopathy within just a few weeks of treatment in several clinical studies, suggested to be the result of its anti-inflammatory impact on the heart in part via plasma reductions of the prolactin 16 kD fragment [507,508]. Others have noted an influence of bromocriptine to reduce congestive heart failure symptoms and to induce regression of left ventricular hypertrophy in peritoneal dialysis patients in small clinical studies [509,510]. In composite, the collection of these clinical studies in cardiometabolic syndrome subjects highlights the real and potential utility of circadian-timed bromocriptine-QR therapy, which re-establishes the natural circadian peak CNS dopaminergic activity and attenuates the treacherous triad, to ameliorate cardiometabolic syndrome and cardiovascular disease risk.

## 11. Conclusions

In conclusion, the biological clock system in vertebrates has evolved mechanisms to anticipate future periods (seasons) of energy demand stress and accordingly prepare/alter whole body metabolism via the neuroendocrine axis to manifest the insulin-resistant state to ensure an adequate glucose supply to the brain and, thus, increase survival potential against the long energy stress period. This seasonal clock system is comprised of seasonally changing temporal interactions of multiple circadian neuronal input activities to the CNS pacemaker clock circuit to thereby regulate its output control of metabolism via the neuroendocrine axis. This circadian metabolic survival system is activated in large part by a decrease in the natural circadian peak of dopaminergic input to the CNS clock system that results in an induction of a CNS hypodopaminergic/elevated SNS tone (often with an overactive HPA axis) to initiate a plethora of cascading downstream neuroendocrine events to induce the obese, insulin-resistant state. This CNS-clock-driven neurophysiology can be triggered by common chronic environmental stresses of Westernized lifestyle (high fat/simple sugar diets, psychosocial stress, altered sleep/wake architecture). In modern man, these chronic “stress” signals “lock” the CNS-clock control of metabolism in the insulin-resistant state chronically (i.e., for years), which over time potentiates insulin resistance syndrome and cardiovascular disease (see Figure 5 and Figure 6). In such pathophysiological states of T2D, circadian-timed administration of the sympatholytic dopamine D2 receptor agonist, bromocriptine-QR, to reinstate the natural circadian peak of CNS dopaminergic activity (and reduce elevated SNS tone) leads to improvement of glucose and lipid dysmetabolism and attenuation of cardiometabolic risk. Prodigious studies across multiple species and several clinical trials demonstrate critically important roles for CNS circadian dopaminergic activity as a major contributing physiological factor in the regulation of cardiometabolic health.

## Data Availability

Not applicable—information provided in references.

## Abbreviations

5-HTP	5-hydroxy-tryptophan
αMSH	α-melanocyte stimulating hormone
Bromocriptine-QR	bromocriptine-quick release
CNS	central nervous system
CRH	corticotropin releasing hormone
CVD	cardiovascular disease
FFA	free fatty acids
HbA1c	hemoglobin A1C
HPA	hypothalamic-pituitary-adrenal
HyperFFAemia	HyperFreeFattyAcidemia
NE	norepinephrine
NO	nitric oxide
NPY	neuropeptide Y
PBMC	peripheral blood mononuclear cells
PVN	paraventricular nucleus
SCN	suprachiasmatic nucleus
RNS	reactive nitrogen species
ROS	reactive oxygen species
S	serotonin
SuMN	supramammillary nucleus
SNS	sympathetic nervous system
T1DM	type 1 Diabetes Mellitus
T2D	type 2 diabetes
TH	tyrosine hydroxylase
VMH	ventromedial hypothalamus
BMAL1	BasicHelix-Loop-HelixARNTLike1
CLOCK	Circadian Locomotor Output Cycles Protein Kaput
CRY1/2	Cryptochrome Circadian Regulator 1/2
DBP	D-Box BindingPAR BZIP Transcription Factor
eNOS	Nitric Oxide Synthase3, Endothelial
IKKαβ	Nuclear Factor NFkappaB Inhibitor Kinase Alpha/Beta
IL-6	Interleukin 6
JAK2	Janus Kinase 2
JNK	Mitogen-Activated Protein Kinase 8
NFIL3	Nuclear Factor, Interleukin 3 Regulated
NFkB	Nuclear Factor Kappa B
PER1/2/3	Circadian Clock Protein PERIOD 1/2/3
PPAR-γ	Peroxisome Proliferator Activated Receptor Gamma
REV-ERBα/β	Nuclear Receptor Subfamily 1 Group D Member 1/2
RORα/β/γ	RAR Related Orphan Receptor A/B/C
SOCS3	Suppressor Of Cytokine Signaling 3
STAT3	Signal Transducer And Activator Of Transcription 3
TLR	Toll-Like Receptor
